# William H. Appleton, Esq.

**Published:** 1899-11

**Authors:** 


William H. Appleton, Esq., the whilom senior member of the house of D. Appleton & Company, of New York City, died at his home, Thursday, October 19, 1899, aged 85 years. The *New York Medical Journal* in referring to Mr. Appleton's death very justly remarks that he was a sterling friend of the medical profession, numbering among it many close friends.

